# A crisis resolution and home treatment team in Norway: a longitudinal survey study Part 3. Changes in morbidity and clinical problems from admission to discharge

**DOI:** 10.1186/1752-4458-6-17

**Published:** 2012-09-11

**Authors:** Stian Biong, Ottar Ness, Bengt Karlsson, Marit Borg, Hesook Suzie Kim

**Affiliations:** 1 Faculty of Health Sciences, Buskerud University College, Box 7053, Drammen 3007, Norway

**Keywords:** Crisis resolution, Mental health home treatment, Mental health services, Community mental health

## Abstract

**Background:**

Crisis resolution and home treatment (CRHT) is an emerging mode of delivering acute mental health care in the community. There is a paucity of knowledge regarding the workings of CRHT in the literature. This is the third paper in a series of three from the longitudinal survey of patients of a CRHT team in Norway, which was aimed at describing the characteristics of patients served, professional services provided, and clinical outcomes. This report focuses on the changes in morbidity and clinical problems from admission to discharge and the length of service.

**Methods:**

The study was a descriptive, quantitative study based on the patient data from a longitudinal survey of one CRHT team in Norway. The participants of the survey, a total of 363 patients, were the complete registration of patients of this team in the period from February 2008 to July 2009.

**Results:**

The findings indicate that the patients´ mental health status improved from admission to discharge, although many patients were discharged with the same mental health symptoms as those present at admission. However, one third of the patients were discharged with no clinically significant mental health problems. The majority of the patients of the CRHT team on the other hand seemed to be those with long-standing mental health problems, who were likely to be in need of continuing mental health care even after the resolution of mental health crises. There is a need for a coordinated system of community-based mental health services for patients with long-standing mental health problems, within which CRHT teams can play a pivotal role in making connections between the crisis-care and the recovery-oriented care. The mean length of service was around 15 days with variations by the clinical problem types, with the patients in the psychosis group having the shortest duration and the patients in the depression group having the longest duration.

## Introduction and background

This paper is the last in a series of three presenting findings from a longitudinal survey studying a crisis resolution home treatment (CRHT) team in Norway [1,2]. It is based on the data of assessment, treatment and outcome registration of a total of 363 patients of the team in the period from February 2008 to July 2009. The focus of this paper is on the changes in morbidity and co-occurring clinical symptoms from admission to discharge.

In line with the emphasis on community based treatment and rehabilitation in mental health care espoused by World Health Organisation [3] and the European policies and in response to the ideology of reform outlined in the National Action Plan for Mental health [4], most Norwegian Community Mental Health Centres (DPS) have organised and developed crisis resolution home treatment teams (CRHT) from 2005 and onward [5]. In the same perspective of the national policies and treatment developments in the UK [4,6], the overall objectives of a multidisciplinary crisis resolution and home treatment (CRHT) team in Norway are to offer comprehensive treatment and support in people’s home environment and prevent hospital admission. A set of guidelines were worked out, based on international experiences with the key service characteristics for CRHT teams being defined as (a) brief responding time, (b) provision of assessment and direct care in the context of home and family, (c) working in partnerships with relevant health and social welfare providers, and (d) assessment and course of action that may include inpatient treatment, home treatment, crisis resolution by the team, and next-level referrals to health and social services [7]. This development has been a part of comprehensive changes in the mental health service system, with an intention to benefit service users and their families. Part of this change has been the call for a shift from the biomedical paradigm orientation to humanistic, person-oriented, and social network oriented mental health services [1].

Surveys in England [6,8-10] and in Norway [2] revealed a great divergence in the workings of CRHT teams in patients served, services provided, caseloads, and taking up the “gate keeping” role. This diversity in specialized community mental health teams may cause challenges for overall service organisation, management, and evaluation. At present, it is difficult to determine if and how such varieties in the structure of CRHT teams impact differently on changes in morbidity and clinical problems from admission to discharge, length of service, and outcomes.

The majority of the patient population of the Community Mental Health Centres in Norway is registered with anxiety disorders and non-psychotic affective disorders [11]. However, the characteristics of morbidity and clinical problems in patients of CRHT teams are not known well. A literature review by Sjølie, Karlsson, and Kim [12] revealed that most of the published articles on CRHT focus on structural issues pertaining to the development of home treatment services and on macro-level outcomes such as cost-effectiveness and admission rates, which may have political, economic and practical implications.

There seems to be a lack of research describing patients´ morbidity, length of service, and outcomes of treatment in CRHT teams [13]. However, one longitudinal survey study has been carried out to investigate the characteristics regarding patients and services provided over a period of 18 months in a CRHT team in Norway at an aggregate level [5]. Extending the findings of the survey by Karlsson et al. [5], the aims of this paper are to: (a) describe the changes in morbidity and co-occurring clinical problems from admission to discharge, (b) examine differences in the length of service related to the changes in morbidity and co-occurring clinical problems, (c) explore key socio-demographic variables which may influence the changes in morbidity and co-occurring clinical problems from admission to discharge, and (d) examine the changes in morbidity and co-occurring clinical problems in relation to the type of services provided by the CRHT team to the patients.

## Methods

### Study design

This is a descriptive, quantitative study based on client data from a longitudinal survey of the patients of one CRHT team in Norway from February 2008 to July 2009.

### Participants

The team is located in an urban district, within a catchment area of 130,000 inhabitants. The team service data covers a total of 363 patients, which make up the entire population that received services from this CRHT team for the period.

### Data collection form

A registration form was used to collect the data, and was based on the Multicentre Study on Acute psychiatry (MAP) [14]. This data form was used to register the CRHT service as a part of a larger study, which included an aggregated data on five CRHT teams in Norway from which a report has been made [2] as well as the patient registration data used in this study. This data set was also used to report the intake information and intake processes, and the service processes in the team in two other reports in a series including this report. The data set addressed the team's actual service in terms of referrals and sources of referrals, patients' personal background, patients' intake and discharge statuses, service duration, services provided, and discharge destination. The unit of the registration was patient for our study, with the data obtained at intake and discharge. The data form consisted of eight sections of which the information in the sections (d), (e), (g) and (h) were used in this paper: (a) intake information including referral sources, (b) personal background information, (c) services received prior to the intake, (d) intake assessment, (e) services provided by the team, (f) types of coordination and cooperation contacts made by the team, (g) discharge assessment, and (h) discharge follow-up recommendations.

### Instruments

#### HoNOS – instrument for mental health status

For assessments of patients' mental health status both at intake and discharge, the Health of the Nation Outcome Scale (HoNOS) [15,16] was used. The HoNOS instrument measures severity of mental health problems in the following 12 categories:

1. Overactive, aggressive, disruptive or agitated behavior

2. Non-accidental self-injury

3. Problems with alcohol or substance abuse

4. Cognitive problems

5. Physical illness or disability problems

6. Problems associated with hallucinations and delusions

7. Problems with depressed mood,

8. Other mental and behavioral problems, including ten items (a = phobia, b = anxiety, c = compulsive behaviors, d = stress/tension, e = dissociative, f = somatoform, g = eating disorder, h = insomnia, i = sexual problem, and j = other problems)

9. Problems with social relationships

10. Problems with activities of daily living

11. Problems with living condition

12. Problems with occupation and activities.

In this instrument each category is rated in the scale of 0 to 4 with zero for "no problem," 1 for “minor problem requiring no action,” 2 for “mild problem but definitely present,” 3 for “moderately severe problem,” and 4 for "severe to very severe problem". For the category #8 that lists 10 items of problems, one major problem is selected for each patient for rating on the same scale of 1 to 4. In this study, ratings on these HoNOS categories are considered mental health morbidity, since the instrument measures the presence and severity of mental health problems specified in these categories.

The scales and subscales of HoNOS [15,16] are HoNOS-Total (HoNOS-T) for summed scores of items #1 to #10, HoNOS-Behavior (HoNOS-B) for summed scores of items #1, #2, & #3, HoNOS-Impairment (HoNOS-I) for summed scores of items #4 and #5, HoNOS-Symptom (HoNOS-S) for summed scores of items #6, #7, & #8, and HoNOS-Social Functioning (HoNOS-SF) for summed scores of items #9 through #12. We constructed a clinical problem grouping from the data, as many patients had more than one problem rated on HoNOS. This was carried out to understand the nature of clinical problems the patients had in a more comprehensive manner than just through the ratings on the HoNOS categories independently. We categorize the HoNOS scores into two levels: “1” as no clinically significant problem (for the scores of 0 to 2), and “2” as clinically significant problem (for the scores 3 and 4) in order to identify co-occurrences of the problems. We also grouped the items of “overactive/aggressive”, “problems with alcohol & drug abuse”, “cognitive problems”, “physical illness or disability problems”, “phobia”, “compulsive behaviours”, “dissociative”, “somatoform”, “eating disorder”, and “other problems” as a consolidated category of “other problems” for this construction. This was done because there were only few patients on these items with the ratings of 3 or 4, except the item on “physical illness or disability” which was viewed to refer to non-mental health problem. The final instrument for the clinical problem type includes seven types labelled as specified in the following:

1. *No clinical problem* Type - No clinically significant problem (no category with the rating of 3 or 4)

2. *Stress only* Type – One problem of stress only (anxiety, stress/tension, or insomnia)

3. *Self-harm* Type - Self-harm only or with other problems including depression

4. *Psychosis* Type - Psychotic problems only or with other problems including depression

5. *Depression* Type - Depression only or with other problems except self-harm and psychotic problems

6. *Single other problem* Type - One other problem only (Of those categorized as *other problems* in the recoding, excluding stress, anxiety, insomnia, self-harm, psychosis, or depression)

7. *Miscellaneous* Type - Two or more other problems

Because there was no case with both psychosis and self-harm occurring together, anchoring the *psychosis* and *self-harm* types independent of each other was possible.

### The global assessment of functioning scales (GAF-S & GAF-F)

In addition to HoNOS, patients were also rated on the Global Assessment of Functioning scales (GAF) both for symptoms (GAF-S) and functioning (GAF-F) at intake and discharge. GAF is a numeric scale (0 through 100) used by mental health clinicians and physicians to rate subjectively the social, occupational, and psychological functioning of adults (e.g., how well or adaptively one is meeting various problems-in-living) [14,17]. Ten ranges of score specify the levels of symptom and functioning ranging from the highest level for no symptoms (GAF-S) and superior functioning in a wide range of activities (GAF-F) to the lowest level for persistent danger of severely hurting self or others (GAF-S) and persistent inability to maintain minimal personal hygiene (GAF-F).

### Data collection procedures

The team members of the CRHT team were trained to use the questionnaire including HoNOS and GAF at the time the team was established. The responsible team member for each patient at admission and discharge filled out the questionnaire. Therefore, all regular professionals of the CRHT team were involved in collecting the data. The data collection was done specifically for this research project. The researchers held quarterly meetings with the professional staff of the team in order to re-train their use of the registration form throughout the data collection period. The data were collected on all patients (363 patients) who went through the intake process for the team during the study period.

### Data analysis

The data were analyzed by the statistical software PASW for Windows version 17.0 for SPSS for descriptive statistics. When comparing two groups the Student´s *t*-test was used for continuous variables and the Pearson´s chi-square test was used for categorical variables. When comparing differences in means in length of service with more than two groups the ANOVA test was applied.

### Ethics

The Regional Medical Research Ethics Committee, Health Region II (South) of Norway and the Norwegian Social Science Data Services on behalf of The National Inspectorate approved this study.

## Results

### The sample and the baseline characteristics

The study group consisted of 363 patients who received services from the CRHT team during the study period of 18 months. The patients in this study were those who became officially registered to receive mental health services by the CRHT team. Individuals upon initial contacts, usually by telephone calls, were assessed by a team member for appropriateness for services by the CRHT team, and only those persons who were judged to be in need of services by the CRHT team were processed for intake registration. Therefore, the data for this study were from the patients who were admitted to the CRHT team. A finding from another data set regarding the total number of referral calls received by this team during 18 months from May 2008 to December 2009 was 1,117 of which 418 patients were admitted to the team. We estimate that a similar number of referral calls would have been received by the team during our study period, suggesting that about one third of the referral calls were admitted to the team. There were no data except the basic demographic information on those individuals who were referred but not admitted to the CRHT team. This means that there were no data on the exact nature of communication at the time specifically regarding the reasons for not admitting the patients. However our knowledge of the team suggests that they would have been told to seek other appropriate services in the community such as clinics or day-care centres. Referrals to inpatient psychiatric emergency units were done after intake and initial assessments.

The study group consisted of more females (65%) than males (35%), and had a majority (72%) in the age group of 26 to 65 years with a significantly lower proportion of the elderly over the age of 65 (6%) in the group compared to the population in the DPS region (16%) and in Norway (15%). The distribution in the marital status is similar to the general adult population in Norway, with 43% being married or in cohabiting status. The results show that only about one quarter of the total group had regular income, and more than one half were on disability/sick pay. A little less than half (40%) were living alone, and about one half of the young adults (ages 26–45) were responsible for childcare while only one third of the middle aged (ages 46–65) had child care responsibilities.

A large proportion (39%) of the patients were referred to the CRHT team either by self or family, and 26% were referred by GPs. Additional 20% were referred by psychiatric professionals or the staff at mental health clinics/daycare centers. The majority of the patients (95%) was referred back to their primary physicians at discharge, and of these 42% was also referred to psychiatric services in the community or to private psychiatric professionals. At discharge 28 patients (8%) were referred to inpatient psychiatric emergency units for admission.

### Changes in morbidity and clinical problem types from admission to discharge

The distributions in the admission and discharge HoNOS categories are shown in Table 1. Patients with the score of 3 or 4 in these categories were included in this table, as the scores above 3 are considered to indicate problems of clinical significance, suggestive of morbidity. The table only includes the HoNOS categories with more than 7 patients (2% of the sample) in each category at this level. Table 1 also shows the numbers of individuals having the same symptoms at admission and discharge in these HoNOS categories. In general, there were fewer numbers of patients with the symptoms at discharge than at admission in these HoNOS categories, with the significant decreases in the insomnia category (11 patients at discharge from 27 at admission), the self-harm category (18 patients at discharge from 32 at admission), the depression category (48 patients at discharge from 81 at admission), and the anxiety category (55 patients at discharge from 71 at admission). The numbers in the categories of alcohol/drug abuse, psychotic problems, and other clinical symptoms remained similar at discharge with those at admission.

**Table 1:** Percent of the total sample with diagnosis in selected HoNOS categories at admission & discharge, and with diagnosis at both admission & discharge

**Selected HoNOS Categories**	**Thos with symptom at admission**		**Those with symptom at discharge**		**Those with same symptom at both admission & discharge**	
	N*	**(%**^**1**^**)**	N*=	**(%**^**1**^**)**	**N=**	**(%**^**2**^**)**
Self-harm	32	(8.82)	18	(4.96)	11	(61.11)
Alcohol & drug abuse	29	(7.99)	26	(7.16)	24	(92.30)
Physical illness	38	(10.47)	32	(8.82)	32	(100.00)
Psychotic problems	27	(7.44)	26	(7.16)	20	(76.92)
Depression	81	(22.31)	48	(13.22)	40	(83.33)
Anxiety	71	(19.56)	55	(15.15)	44	(80.00)
Stress	67	(18.46)	37	(10.19)	29	(78.38)
Insomnia	27	(7.44)	11	(3.03)	11	(100.00)
Other clinical symptoms	37	(10.19)	33	(9.09)	27	(81.82)
Problems with social relations	67	(18.46)	52	(14.33)	46	(88.46)
Problems with ADL	28	(7.71)	25	(6.89)	14	(56.00)

^1^Percent of the total sample (n = 363); ^2^Percent of those with the diagnosis at discharge in the same category; *Many patients were in more than one HoNOS categories.

Large proportions (around 80% in general) of those patients who had these symptoms at admission also had the same symptoms at discharge as shown in Table 1. However, except for the categories of physical illness and insomnia, there were patients who acquired new symptoms at discharge that were different from those at admission. Most significantly, while only 11 patients (61% of the total in this category) were assessed to have self-harm as the symptom at both admission and discharge, there were also 7 patients who were assessed to have self-harm as the symptom at discharge although they did not have it at admission. These indicate that 21 patients who were admitted to the service with self-harm as the symptom did not have it at discharge, while 7 other patients who did not have the symptom at admission had it at discharge.

The data in the category of problems with social relations show that nearly one fifth of the patients had problems with social relations at admission and the majority (88%) remained to have the problem at discharge. On the other hand, only a little more than half of those with problems with activities of daily living at admission still had the problem at discharge, while there were other patients who ended up with the problem at discharge while they did not have it at admission (11 patients).

**Figure 1:**
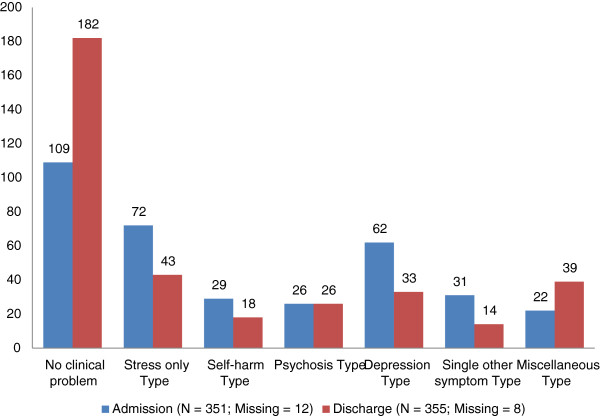
Distribution in the clinical problem types at admission and discharge.

Figure 1 shows the distribution in the clinical problem types at admission and at discharge. The greatest increase is in the *no clinical problem* type in which there was 51% of the total (182 patients) at discharge compared to 31% (109 patients) at admission. Accordingly, there were decreases in other types from admission to discharge except in the *psychosis* type and the *miscellaneous* type. There were decreases in the *stress only* type from 21% at admission to 12% at discharge, the *depression* type from 18% at admission to 9% at discharge, the *single other problem* type from 9% at admission to 4% at discharge, and the *self-harm* type from 8% at admission to 5% at discharge. The *psychosis* type remained at the same level (7.4% at admission and 7.3% at discharge) while there was an increase in the *miscellaneous* type from 6% at admission to 11% at discharge.

**Figure 2:**
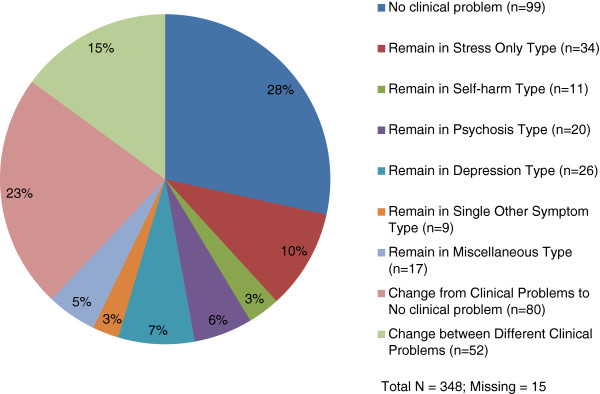
Distribution in the change types from the admission clinical problem types to the discharge clinical problem types.

Figure 2 shows the changes from admission to discharge in the clinical problem types. Ninety nine patients (28%) remained the same in the *no clinical problem* type both at admission and discharge, while 80 patients (23%) who were in various clinical problem types other than the *no clinical problem* type at admission ended up in the *no clinical problem* type at discharge. A total of 117 patients (34%) were in the same clinical problem types both at admission and discharge, with 34 patients (10%) in the *stress only* type, 11 patients (3%) in the *self-harm* type, 20 patients (6%) in the *psychosis* type, 26 patients (7%) in the *depression* type, 9 patients (3%) in the *single other problem* type, and 17 patients (5%) in the *miscellaneous* type.

Table 2 shows the means in HoNOS scales, and in GAF-S and GAF-F scales at admission and discharge. For all scales, there were significant improvements in the levels of severity in mental health problems at discharge compared to those at admission. For HoNOS scales, the mean values were lower at discharge than at admission, indicating decreases in the severity levels with mental health problems. Both GAF-S and GAF-F scales increased at discharge, showing significant changes from admission to discharge, also indicating improvement in the levels of the general mental health symptom experiences and mental health functioning measured by these instruments.

**Table 2:** Means and SD for HoNOS scales, GAF-S & GAF-S at admission and discharge, and the results of comparison between the means at admission and at discharge

**Scales**	**Time**	**Mean (SD)**	**SE Mean**	**Paired Difference**				**t value**
				**Mean (SD)**	**SE Mean**	**95% CI**		
						**Lower**	**Upper**	
HoNOS-T	Adm	9.76 (4.69)	.25	1.51 (3.12)	.17	1.18	1.84	t = 9.04* (df = 347)
	Dis	8.25 (5.11)	.27					
HoNOS-S	Adm	4.91 (1.79)	.09	.70 (1.50)	.08	.54	.86	t = 8.83* (*df* = 358)
	Disc	4.21 (2.07)	.11					
HoNOS-B	Adm	1.78 (1.83)	.10	.50 (1.38)	.07	.35	.64	t = 6.73* (*df* = 352)
	Disc	1.28 (1.69)	.09					
HoNOS-I	Adm	1.11 (1.42)	.08	.16 (.67)	.04	.09	.23	t = 4.54* (*df* = 360)
	Disc	.95 (1.36)	.07					
HoNOS-SF	Adm	2.45 (2.52)	.13	.199 (1.39)	.07	.056	.343	t = 2.73* (*df* = 360)
	Disc	2.26 (2.52)	.13					
GAF-S	Adm	48.33 (10.37)	.55	−4.72 (8.55)	.45	−5.61	−3.83	t = −10.43* (*df* = 356)
	Disc	53.05 (12.18)	.65					
GAF-F	Adm	48.14 (13.18)	.70	−3.57 (8.18)	.43	−4.43	−2.72	t = −8.26* (*df* = 356)
	Disc	51.71 (14.23)	.75					

*p significant at .05; Missing values for each scale category were different ranging from 2 to 15 evident in the degrees of freedom for each comparison; "Adm" stands for the assessment at admission, and "Disc" stands for the assessment at discharge.

Table 3 shows the GAF-S and GAF-F at discharge and the GAF-S and GAF-F change scores from admission to discharge according to the change categories in the clinical problem types. There were significant differences in the mean discharge GAF-S and GAF-F scores by the change categories in the clinical problem types with the *no clinical problem* type at both times with the highest mean scores in GAF-S (59.30) and GAF-F (60.32) and the *psychosis* type at both admission and discharge with the lowest mean scores in GAF-S (31.26) and GAF-F (31.05). The mean change scores for GAF-S and GAF-F for the study sample were positive at 4.81 and 3.62 respectively, indicating positive gains. The group that was composed of those in various clinical problem types as the diagnoses at admission but in the *no clinical problem* type at discharge had the highest mean positive change score in GAF-S (12.06), and GAF-F (9.56), followed by the group in the *no clinical problem* type both at admission and discharge (the mean of 5.40 for GAF-S and of 4.03 for GAF-F). The group that remained within the *self-harm* type at both admission and discharge had the least change with the negative mean change scores for GAF-S (−0.55) and GAF-F (−0.82).

**Table 3:** Means for GAF-Symptom and GAF-Function at discharge and mean change scores in GAF-Symptom and GAF-Function from admission to discharge in the change categories in the clinical problem types from admission to discharge

**Type of change in clinical problem categories at discharge**		**GAF at discharge**		**Change in GAF scores from admission to discharge**	
		**GAF-Symptom**	**GAF-Function**	**GAF-Symptom**	**GAF-Function**
*No clinical problem* at both admission & discharge	Mean (SD) N = 99	59.30 (9.316)	60.32 (11.620)	5.40 (7.311)	4.03 (6.713)
*Stress only* type at both admission & discharge	Mean (SD) N = 34	52.24 (9.303)	49.65 (11.990)	0.706 (4.191)	1.09 (5.201)
*Self-harm* type at both admission & discharge	Mean (SD) N = 11	38.27 (13.951)	40.09 (16.525)	- 0 .55 (3.236)	- 0.82 (5.845)
*Psychosis* type at both admission & discharge	Mean (SD) N = 19	31.26 (8.451)	31.05 (6.737)	0.28 (5.686)	- 0.22 (3.639)
*Depression* type at both admission & discharge	Mean (SD) N = 26	45.69 (7.594)	41.69 (7.817)	1.12 (3.681)	1.00 (4.079)
*Single other clinical problem* type at both admission & discharge	Mean (SD) N = 8	58.63 (11.211)	56.63 (11.673)	1.88 (2.748)	0.75 (2.121)
*Miscellaneous* type at both admission & discharge	Mean (SD) N = 17	48.59 (6.577)	42.24 (7.429)	1.82 (5.876)	2.00 (6.195)
Clinical problems at admission (all types) &*no clinical problem* type at discharge	Mean (SD) N = 79	59.15 (9.876)	57.78 (13.626)	12.06 (9.510)	9.56 (10.539)
Various clinical problem types at admission to different clinical problem types at discharge	Mean (SD) N = 51	48.24 (10.594)	45.33 (10.465)	1.24 (9.616)	- 0.06 (7.809)
Total	Mean (SD) N = 344^†^	53.13 (12.343)	51.81 (14.412)	4.81 (8.681)	3.62 (8.224)
Simple Effects F: *df* (8, 336)		29.111** (p < .01)	24.648** (p < .01)	14.597** (p < .01)	10.031** (p < .01)

**p significant at < .01; ^†^Missing = 19.

### Length of service related to changes in clinical problem types

The mean length of service for the 363 patients was 15.4 days. Table 4 shows differences in the mean length of service according to the admission clinical problem types and the changes into the discharge clinical problem types. In terms of the admission clinical problem types, the shortest mean length of service was in the *psychosis* type (9.5 days) and the *miscellaneous* type (9.6 days), while the *depression* type at admission had the longest mean length of service of 23.3 days. With regards to the change types from admission to discharge, those patients who remained with the same clinical problems had shorter service duration, with the group means ranging from 5.6 days to 15.0 days. The shortest length of service was in the group that stayed in the *psychosis* type (5.6 days) followed by the group that stayed in the *self-harm* type (6.8 days). The patient group that ended up with different clinical problems at discharge from their admission problems had longer mean lengths of service ranging from 20.6 days to 27.7 days. Of those groups that shifted to the *no clinical problem* type, the *depression* type had the longest mean length of service at 32.6 days followed by 22.3 days by the *psychosis* type.

**Table 4:** Mean length of service duration by the admission clinical problem type and the discharge clinical problem type

**Admission Clinical Problem Type**	**Discharge Clinical Type**						**Total**	
	**The same type**		**No clinical problem type**		**Other clinical problem types**			
	**N =**	**Mean (SD)**	**N =**	**Mean (SD)**	**N =**	**Mean (SD)**	**N=**	**Mean (SD)**
*No clinical problem* type	99	14.95 (16.83)	-	-	7	16.71 (14.22)	106	15.07 (16.06)
*Stress only* type	34	8.82 (9.87)	31	16.71 (12.41)	7	22.86 (19.54)	72	13.58 (12.93)
*Self-harm* type	11	6.82 (9.87)	10	17.40 (7.59)	8	23.75 (17.74)	29	15.14 (13.50)
*Psychosis* type	20	5.60 (6.94)	6	22.33 (10.71)	-	-	26	9.46 (10.55)
*Depression* type	26	12.58 (13.14)	25	32.56 (24.15)	11	27.73 (32.82)	62	23.32 (23.76)
*Single other clinical problem* type	9	11.33 (15.75)	5	14.80 (12.05)	17	20.59 (20.87)	31	16.97 (18.32)
*Miscellaneous* type	17	8.35 (17.96)	3	7.33 (8.39)	2	24.00 (11.31)	22	9.64 (16.74)
Total^1^	216	11.75 (14.77)	80	21.70 (17.86)	52	22.50 (21.78)	348	15.64 (17.39)
Simple Effects F for Service Duration by Admission Clinical Problem Type: *df* (6, 341)							F = 3.352** (p = .003)	

^1^Missing = 15; **p significant at .01.

In further examinations of the data, it was found that the short mean length of service for the *psychosis* type at both admission and discharge was due to the fact that 10 patients of this group were discharged to inpatient psychiatric emergency units with a mean length of service of 2.6 days. Similarly, of those patients in the *stress only* type at both admission and discharge (a total of 34 patients), 4 patients who were discharged to inpatient psychiatric emergency units had a mean length of service of 2.0 days. There were 6 patients who were discharged to inpatient psychiatric emergency units in the group that changed from various clinical problem types at admission to other clinical problem types at discharge, whose mean length of service was 9.6 days compared to the mean (24.1 days) of the rest of this group.

### Demographic variables in relation to changes in morbidity and clinical problem types

There was only one socio-demographic variable (the income source) that gave statistically significant results in relation to the change types in the clinical problem groupings from admission to discharge. None of the socio-demographic variables was significantly associated with the changes in HoNOS categories from admission to discharge. Table 5 shows the distribution in the change types in the clinical problem groupings from admission to discharge by income source. Patients remaining in the *depression* type both at admission and discharge and those remaining in the *miscellaneous* type both at admission and discharge were more likely to be on sick/disability pay compared to other groups, while those who were in the *no clinical problem* type both at admission and discharge, those in the *self-harm* type at both times, and those in other clinical problem types at admission but in the *no clinical problem* type at discharge were more likely to have regular income when compared to other groups. The income source type was also significantly different in the means of GAF-S change scores (F = 5.556 with p = .004): The mean for the regular income group was 6.795 (SD = 8.983) compared to the mean of 3.806 (SD = 8.040) for the sick/disability pay group and the mean of 3.027 (SD = 8.830) for the other income group. Although the regular income group had a higher mean decrease in the HoNOS-T from admission to discharge, meaning an increase in mental health status, compared to the sick/disability group, this was not statistically significant.

**Table 5:** Distribution in income source types by the change category in the clinical problem types from admission to discharge

**Change Categories in the Clinical Types from Admission to Discharge**		**Income Source Type**			
		**Regular Income**	**Sick/disability Pay**	**Others**	**Total**
*No clinical problem* type at both admission & discharge	N (%)	46 (46.9)	42 (42.9)	10 (10.2)	98 (100.0) (28.3)
*Stress only* type at both admission & discharge	N (%)	9 (26.5)	22 (64.7)	3 (8.8)	34 (100.0) (9.8)
*Self-harm* type at both admission & discharge	N (%)	5 (45.5)	6 (54.5)	0 ( − )	11 (100.0) (3.2)
*Psychosis* type at both admission & discharge	N (%)	5 (25.0)	12 (60.0)	3 (15.0)	20 (100.0) (5.8)
*Depression* type at both admission & discharge	N (%)	6 (24.0)	18 (72.0)	1 (4.0)	25 (100.0) (7.2)
*Single other clinical problem* type at both admission & discharge	N (%)	3 (33.3)	5 (55.6)	1 (11.1)	9 (100.0) (2.6)
*Miscellaneous* type at both admission & discharge	N (%)	1 (5.9)	13 (76.5)	3 (17.6)	17 (100.0) (4.9)
Clinical problems at admission (all types) &*no clinical problem* type at discharge	N (%)	34 (42.5)	42 (52.5)	4 (5.0)	80 (100.0) (23.1)
Various clinical problem types at admission to different clinical problem types at discharge	N (%)	8 (15.4)	34 (65.4)	10 (19.2)	52 (100.0) (15.0)
Total	N (%)	117 (33.8)	194 (56.1)	35 (10.1)	346^†^ (100.0)
*χ*^2^ Results	*χ*^2^ = 34.889** (*df* = 16; p = .004)				

^†^Missing = 17; **p significant at .01.

### Types of services received and changes in morbidity and clinical problem types

In response to the patients' needs for mental health care, the CRHT team provided direct care by individual treatment meetings mostly held at patients' homes and group treatment meetings held at the team's office. An individual treatment meeting refers to a face-to-face meeting between a member of the team and a patient in addressing the patient’s crises and problems. Although there may have been telephone contacts between the patients and the professionals in addition to these individual counselling meetings, we did not collect data on telephone contacts. Individual professional counselling by one of the members of the CRHT team was the main type of treatment for the patients, and the amount of individual professional services received by the patients can be estimated by the service duration on the team, as most of the patients received one-to-one professional services at least one to two times per week. The majority of the patients were seen by psychiatric nurses (95%) and social workers (76%), while one fourth of the patients met with clinical psychologists and 12% were seen by a psychiatrist. However, the patients often had individual treatment meetings with more than one member of the team. About one fourth of the patients (23%) were seen only by psychiatric nurses, while nearly one half (46%) were seen by both a psychiatric nurse and a social worker. On the other hand, about one third of the patients (31%) were seen by a psychiatrist and/or a clinical psychologist in addition to nurses and/or social workers. There was no difference in the type of professionals and the combination type of professionals providing individual treatment meetings in terms of the patients' mental health status in the HoNOS categories, the clinical problem types, HoNOS scales, and GAF scales at admission.

We examined the changes in HoNOS categories and the changes in the clinical problem types from admission to discharge in relation to the different combination of individual treatment meetings with the patients. The data showed that there was no systematic difference in the changes from admission to discharge in mental health status according to the types of professionals providing individual treatment services to the patients in general.

The CRHT team was also involved in coordination/cooperation activities involving healthcare providers and service units external to the team on behalf of the patients. However, coordination activities for the patients had no association with the changes in the HoNOS categories and the changes in the clinical problem types from admission to discharge.

In addition to individual treatment meetings, the CRHT team provided various group treatment meetings for the patients such as family/network meetings involving patients and their families or network members with a team member assigned to specific patients (family/network meeting), meetings of the team members together with specific patients to address patients' problems as a team (team treatment meeting), group therapy meetings involving patients with similar clinical problems with a member of the team (group therapy meeting), and group activity meetings. Family/network meetings and team treatment meetings were held for specific patients as determined by the team members, which could happen more than once per patient, while group therapy meetings and group activity meetings were on-going at the CRHT setting in which the patients were invited to participate. Thirty seven percent (37%) of the patients received family/network meetings, and 21% of the patients were involved in team treatment meetings. Only a few cases were involved in group therapy meetings or in group activity meetings. About one half of the patients (52%) did not receive group treatment meetings, while one fourth (26%) were in family/network meetings only and about one fifth (11%) were in team treatment meetings only, while another 11% received both family/network meetings and team treatment meetings.

**Figure 3:**
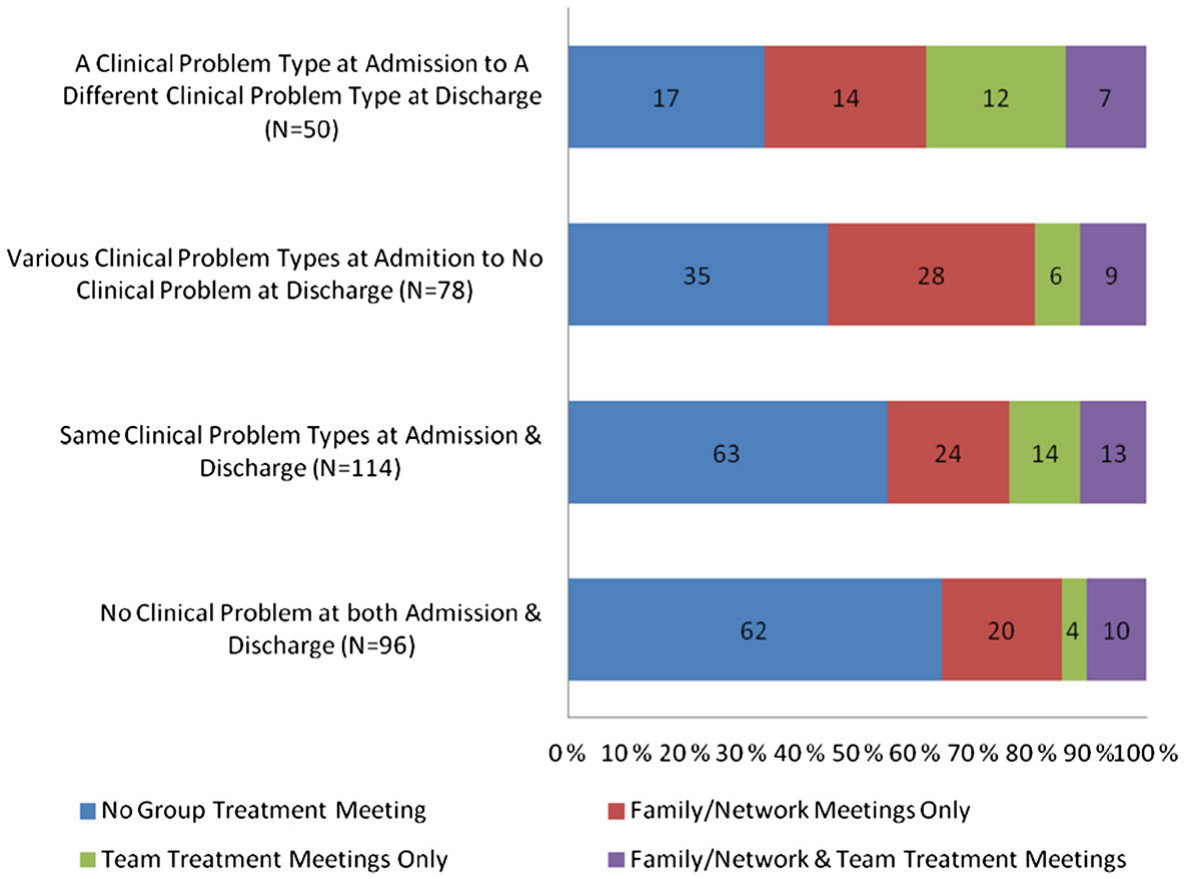
Distribution in the type of change in clinical problem types from admission to discharge by the type of group treatment meetings held with patients.

Figure 3 shows the distribution in the types of group treatment meetings held for the patients according to the changes in the clinical problem types from admission to discharge. This distribution was statistically significant (*χ*^2^ = 25.570, *df* = 9, & p = .002). “No group treatment meeting” was the highest in the *no clinical problem* group both at admission and discharge (65%) and the lowest in the group that had one type of clinical problem at admission but had a different clinical problem at discharge (34%). On the other hand, the percentages receiving family/network meetings only ranged from 21% in the *no clinical problem* group both at admission and discharge to 36% in the group that had no clinical problem at discharge although they had specific clinical problem at admission. The patients who were discharged with different clinical problems were most likely to receive the combination of family/network and team treatment meetings. In general, a similar proportion of the groups received team treatment meetings ranging from 10% to 14%.

Table 6 shows the mean change scores in HoNOS-T, GAF-S, and GAF-F according to the type of group treatment meetings the patients received. The highest positive changes were in the group with family/network meetings only in HoNOS-T (a high negative change score indicating improvement in mental health status), GAF-S, and GAF-F. The lowest gains were evident in the group having a combination of treatment meetings for HoNOS-T (the smallest negative change score) and GAF-S, while the group with no group treatment meeting had the lowest gain in GAF-F. There were significant group differences in the HoNOS-T between the family/network meeting only group and the combination of family/network and team treatment meetings (−2.05 versus −0.42), and in GAF-F between the group with no group treatment meeting and the group with family/network meeting only (2.66 versus 5.75).

**Table 6:** Means and SD in the change scores for HoNOS-T, GAF-S, & GAF-F from admission to discharge by the type of group treatment meetings held

**Group treatment meeting type**	**HoNOS-T Change Score**		**GAF-S Change Score**		**GAF-F Change Score**	
	**N=**	**Mean (SD)**	**N=**	**Mean (SD)**	**N=**	**Mean (SD)**
No group treatment meeting	177	−1.49 (2.79)	184	4.40 (7.65)	184	2.66^b^ (6.63)
Family/network meetings only	86	−2.05^a^ (3.34)	89	6.70 (8.90)	89	5.75^b^ (8.85)
Family/network meetings & team treatment meetings	36	−0.42^a^ (3.45)	35	2.63 (11.41)	36	3.57 (10.79)
Team treatment meetings only (No family/network meeting)	391	−1.60 (3.49)	39	3.74 (9.00)	39	3.36 (10.55)
Total	338^1^	−1.53 (3.11)	347^2^	4.73 (8.62)	347^2^	3.63 (8.27)
Simple Effects F:	F = 2.369 (p = .071) *df* (3, 334)		F = 2.531 (p = .057) *df* (3, 344)		F = 2.849* (p = .037) *df* (3, 344)	

^1^Missing = 25, ^2^Missing = 16; *p significant at .05; The superscripts, a and b, stand for the significant mean differences between two groups at the .05 level.

## Discussion

In this study we investigated the patients of one CRHT team with regards to (a) the changes in morbidity and clinical problems from admission to discharge, (b) differences in length of service related to changes in morbidity and clinical problems, (c) socio-demographic variables that might influence the differences in the changes in morbidity and clinical problems from admission to discharge, and (d) whether or not the services received by the patients had any impact on the changes in morbidity and clinical problems.

### Changes in morbidity and clinical problem types from admission to discharge

Although there are several studies in the literature reporting diagnoses and morbidity of patients with the services by community mental health care teams such as CRHT teams at admission or at discharge, there is no study that reports changes in morbidity or diagnosis from admission to discharge in relation to CRTH services. The admission morbidity in this study is in line with the results from another study of CRHT teams in Norway from 2005–2008 [14]. The study reported that the majority of patients had either mood/affective symptoms (32%) or neurotic/stress related symptoms (22%) and only few had psychotic symptoms (13%). Barker et al. [18] in their study of CRHT teams in Scotland also found 40% of the patients with depression or bipolar disorder, 15% with schizophrenia, and 18% with personality disorder as their discharge diagnoses. The findings from Hasselberg et al. study [14] regarding the means for HoNOS-Total, GAF-S and GAF-F at admission are similar to our findings, suggesting that the patients of this CRHT team were similar in mental health status with CRHT patients in Norway in general. The admission HoNOS-Total mean in our group is the same as the one found in the study by Johnson et al. [19] in UK. The general picture that emerges both from our data and the literature regarding the mental health status of CRHT patients is that patients tend to have long-standing mental health problems and are at a moderate level of mental health distress.

There was a high increase in the number of patients with no discernible mental health symptoms at discharge from admission (109 patients at admission to 182 at discharge). It indicates that one half of the patients were discharged from the CRHT team services without apparent mental health symptoms. This also indicates an improvement in mental health states in about one fifth of the patients. However, about one third of the patients were admitted with no discernible mental health symptoms measured by HoNOS, suggesting that their needs for the services by the CRHT team may have been assessed by other methods.

In addition, there also were decreases in the numbers of patients from admission to discharge in most of the HoNOS symptom categories and the clinical problem types. These results suggest that there were significant changes in the patients' mental health problems from admission to discharge. However, the decreases were more dramatic in the categories of self-harm, depression, stress, anxiety, & insomnia. The improvements were mostly in relation to transient mental health problems, rather than in long-standing, persistent problems such as alcohol/drug abuse, physical illness, & psychotic problems. Improvement in mental health status at discharge was also evident indicated by the significant differences in the HoNOS and GAF scales between those at admission and those at discharge.

However, our findings that around 80% of the patients in each HoNOS category had the same mental health symptom both at admission and discharge except for the category of self-harm suggest that mental health symptoms specified in HoNOS may tend to be long-term in nature. One third of the patients also remained with the same clinical problem types at admission and discharge, affirming the long-term nature of mental health problems. It is possible that even if mental health crises were to have been resolved in these patients through the services, such mental health symptoms and problems may remain as underlying issues in these patients. It also suggests that patients continuing to have such mental health symptoms and problems may be more vulnerable to mental health crises. In addition, those patients who remained within the *self-harm* type and the *psychosis* type had the lowest mean scores on GAF-S and GAF-F, and had low changes in these scales from admission to discharge, suggesting an insidious nature of these mental health problems.

In general, the improvement in mental health status and the positive changes in the presence of mental health problems from admission to discharge suggest a possible role played by the CRHT team in assisting the patients with their mental health issues. However, since our study is only descriptive in its design, such improvement cannot be inferred to the services by the CRHT team directly. Furthermore, it is possible that the changes could result from the passage of time. In addition, many patients were discharged from the CRHT team with clinically significant mental health problems. There may be several reasons for this. Basically, the team may have focused on crisis resolution in line with the goal of its establishment rather than working on general mental health problems, or that since the majority of the patients had long-standing mental health problems, such mental health problems may continue to exist in these patients. The findings also suggest that the HoNOS instrument may not capture people's mental health status specifically in relation to crisis adequately.

### Differences in length of service related to admission clinical problem types and changes in clinical problem types

The mean length of service for the patients of the CRHT team of 15 days is in line with the recommendation for the functioning of CRHT teams in Norway, which is 30 days as the standard. It is somewhat shorter than the finding of 3 weeks for two teams in Scotland by Barker et al. [18]. The length of service according to the admission mental health status showed that the *depression* type had the longest duration of service, and the groups in the *psychosis* type and the *miscellaneous* type had the shortest service duration. This suggests that *depression* type of clinical problems may represent a complex mental health problem requiring longer services than other types. In addition, the patients who had the intake assessment of depression or psychosis but ending up with no clinical problem at discharge had longer service duration than other groups. This suggests that such patients may have required longer period of time to recover to a status appropriate for discharge than other patients. The findings that the groups that ended up with different clinical problems at discharge from those at admission had longer service durations are intriguing. It is possible that these patients experienced an emergence of other mental health issues during the course of service, which required longer service duration.

The patients who were discharged to inpatient psychiatric emergency units had shorter mean lengths of service, especially those who remained with the same diagnoses in the *psychosis* type, the *stress only* type, and various clinical problem types. These results suggest that the decisions to refer patients to inpatient care seemed to have been made rather expediently following the intakes. Although the proportion of patients discharged to inpatient care was small, the finding that over one third of those discharged for inpatient care were assessed to have psychotic problems both at admission and discharge is significant. It suggests that mental health crises associated with psychosis may be more likely to require inpatient care than other types of mental health problems.

### Socio-demographic variables in relation to the change in clinical problem types from admission to discharge

Our findings indicate that socio-demographic variables such as age, gender, marital status, and living situation are not in general related to the changes in morbidity and clinical problems from admission and discharge. However, in our study we reported that older adults and males were less likely to use the CRHT service, suggesting that age and gender, although having some impact on service-utilization, do not seem to differentiate clinical changes through the services of the CRHT team.

However, a study of patients in community mental health centres in Norway found males, single, and living alone were more likely to have comorbidity of psychiatric disorders and substance abuse disorders, compared to users without substance abuse disorders [11]. Topor et al. [20] also noted the findings of more severe mental health problems in people living alone, who are unemployed, and with less disposable income compared with the general population. In our study, the result of self-selection in seeking help from the CRHT team may be the reason for the lack of significant differences in the changes in clinical problem types from intake to discharge by gender. Our findings that higher proportions of the patients who remained in the *depression* type and *miscellaneous* type both at admission and discharge were those on sick/disability pay indicate the long-term nature of mental health problems in this group. In addition, the patients on sick/disability pay had lower gain scores in GAF-S and GAF-F than those on regular income, suggesting that it may be more difficult to improve in mental health status for patients on sick/disability, who were more likely to have long-standing mental health problems. It appears that it is not necessarily the income source that differentiates the nature of changes but the long-term nature of mental health problems patients have.

### Types of services received and changes in morbidity and clinical problem types

We found that the changes in mental health status were not related to the type of professionals providing individual treatment meetings. This result may mean (a) that individual treatment meetings by different professionals were not in response to mental health status or clinical problems but in order to address presenting mental health crises, which may not be directly related to mental health status, (b) that different combinations of individual treatment meetings by professionals in CRHT teams may have a similar impact on changes in mental health status in general, or (c) that the instruments used in this study to measure mental health status were not sensitive to differentiate the individual treatment protocols applied by the team.

The results that there were some differences in the changes in mental health status and clinical problem types according to the types of group treatment meetings provided to the patients provide some insights regarding the workings of the CRHT team. The findings that the group receiving family/network treatment meetings only had the highest improvements in all mental health scales suggest a possibility of a higher potential for improvement in patients receiving these meetings in comparison to others. In addition, the group receiving both family/network and team treatment meetings had the lowest mean changes in HoNOS-T and GAF-S, suggesting that this group may have had more complex mental health issues. However, the effects of services on the nature of changes in mental health status are not clear.

The results of our study are very specific to the research site and the study design is descriptive. However, the local experiences provide insights into the workings of CRHT teams and raise some important questions which can have impact on various types of considerations for policy and design of services for CRHT teams.

### Methodological considerations

There are several shortcomings related to this study. The variables regarding the level of mental health problems at admission and at discharge were based on the team members´ subjective assessment of the patients´ situations. This could have implications with regard to registration or recall bias, even though training was given throughout the study period. As a study of one single unit, albeit a longitudinal one, the sample cannot be claimed for representativeness and external validity. The researchers´ construction of the clinical problem types might be imperfect and other constructions could have given other results. Another question is whether or not the identified changes from admission to discharge, differences in length of service and the influence of socio-demographic variables in relation to clinical problem types are *clinically* important to CRHT teams.

## Conclusion

The aims of our study were addressed in the results that describe the changes in mental health problems, and examine the relationships between the changes in mental health status to the length of service, socio-demographic variables, and service provisions by the team. Generally, based on the staffs´ registrations, the patients´ mental health status seem to have improved from admission to discharge. However, many patients had the same types and levels of problems at discharge as those with which they entered the service. This may indicate that HoNOS as an instrument of assessment is not sensitive enough to detect fine changes, or that mental health problems that are assessed by HoNOS, such as depression, psychosis, self-harm, etc., do not change with a short-term service that is oriented to crisis resolution. It may also mean that patients of CRHT teams tend to have long-standing mental health problems, which cannot be resolved by the teams’ services of a short duration. There is a need for a coordinated system of community-based mental health services for patients with long-standing mental health problems, within which CRHT teams can play a pivotal role in making connections between the crisis-care and the recovery-oriented care.

Overall, the mean length of service was 15 days, differing by the clinical problem types, with an indication that the types of mental health problems may have some impact on the length of service by CRHT teams. The patients on sick/disability pay were least likely to improve from admission to discharge in general. This might indicate that the patients in this group have long-term mental health problems as well as a variety of social and economical problems, for which the CRHT team may have been able to address the crises situations only. Probably, the CRHT team may be able neither to encounter and dealt with the complex social and material conditions such a person is in nor treat underlying conditions within the service structure of the CRHT team within a short duration. This suggests the nature of mental health crises to be complexly intertwined with existing mental health problems. This also means that the use of HoNOS as an assessment tool may be inadequate in assessing patients´ actual needs in terms of mental health crisis.

### The findings suggest that several issues need to be explored further in future studies

What is the nature of mental health crises for which patients seek or are referred to for the services by CRHT teams? In this study, it was not possible to determine the nature of mental health crises the patients experienced, for which the team's services were sought. There is a need to address this either by establishing a protocol for the determination of mental health crisis that goes beyond the use of HoNOS in order to examine this issue quantitatively or by carrying out in-depth qualitative studies to look into team members' assessment of mental health crises. Such studies will provide knowledge about not only the nature of crises but also how patients respond to the services by CRHT teams.

In this study, the majority of the patients were diagnosed with depression and long-term mental health problems. There is a need to examine in future studies the most appropriate role CRHT teams can play in addressing these mental health problems.

There is also a need for in-depth studies examining how contextual and socio-economic aspects of patients' social situations affect both the experience of mental health crisis and the process of recovery from mental health crises.

Although this study was done with one CRHT team, the findings provide the beginning base with which we can develop bench-marks for services by CRHT teams. As the institution of CRHT teams or of similar service models is increasing rapidly, it is necessary to gain in-depth understanding about the workings of such teams locally, nationally, and internationally.

## Competing interest

The authors declare that they have no competing interests.

## Authors’ contributions

All authors were actively involved in the research project and contributed to all aspects in the preparation of the manuscript. All authors read and approved the final manuscript.
